# Seeing, Being Seen and Being Able to See Dyslexia in English Schools: Parent and Teacher Perspectives

**DOI:** 10.1002/dys.70003

**Published:** 2025-03-24

**Authors:** Angela M. Thompson, Clare Wood, Ian K. Thompson, Julia M. Carroll

**Affiliations:** ^1^ Centre for Global Learning, Education and Attainment Coventry University Coventry UK; ^2^ Psychology Department Nottingham Trent University Nottingham UK; ^3^ Independent Researcher; ^4^ School of Education University of Birmingham Birmingham UK

**Keywords:** Dyslexia Diagnosis, Inclusion, Parent Support, SEND, SpLD

## Abstract

In this study, we present an analysis of interviews with parents and teachers in order to understand the lived experience of families and teachers trying to support children with written language difficulties. Within these accounts, the value of a dyslexia diagnosis is examined in practice. Nineteen parents and 20 educators were interviewed, including four parents who completed additional interviews 4 years after their original accounts. Our analysis indicated that dyslexia is rendered largely invisible in our sample of English schools, with staff frequently reluctant to fully acknowledge it. The analysis suggests mechanisms that appear to underpin successful support or harm to students. An important feature was the role and recognition of diagnosis in increasing the visibility of children's needs. Without recognition and understanding, support was inconsistent and ineffectual.

Literacy, the ability to understand and produce written language, is an important sociocultural tool (e.g., Perry 2012). Literacy allows us to move beyond direct but transitory oral interpersonal communication and to access and create lasting contributions to society and culture (Freire 1983). Within that context, a systematic difference in the capacity to become literate has broader impacts with respect to societal participation, as well as immediate impacts on the ability to function day‐to‐day. However, there are questions about how visible and impactful those difficulties are within the education system.

## 1 The Nature and Diagnosis of Dyslexia

One form of restriction in acquiring literacy is termed dyslexia. A recent consensus definition (Carroll et al. 2025) indicates that dyslexia is primarily a set of processing difficulties that affects aspects of literacy attainment, despite the educational opportunity to learn to read and spell. These processing difficulties can include phonological processing, working memory and processing speed. Difficulties in reading fluency are a key marker of the disorder. These difficulties are rooted in the cognitive processes that underpin learning to read and write, and other skills and behaviours that are dependent on those same disrupted processes (e.g., phonological memory) are likely to be impacted too (Roitsch and Watson 2019).

The unexpected nature of the difficulty, the diversity in strengths and difficulties experienced by the learner, as well as the ‘unconventional intellect when confronted with problems and situations’ (Macdonald 2019, 18) are features of the lay‐narrative associated with dyslexia, along with reading and writing difficulties (see also Rooke 2017).

There is controversy about how dyslexia should be recognised and diagnosed (Evans 2020; Kirby 2020; Protopapas 2019; Snowling et al. 2020). In particular, Elliott (Elliott and Grigorenko 2014; Elliott 2020) argues that there is no meaningful way of identifying a dyslexic subgroup within the larger pool of poor readers, and even if there were, no interventions are differentially appropriate for these two groups. Hence, he argues that there is no value in the diagnostic assessment of dyslexia. This view has been taken on by some local education authorities in the UK, both implicitly and explicitly (Bodkin 2019).

The view that dyslexia has a lack of diagnostic utility partially aligns with the Special Educational Needs and Disability (SEND) Code of Practice (Department of Education and Department of Health 2015). This document, which advises on access and support for children with SEND in England, emphasises support according to need, rather than diagnosis. This has the laudable aim of reducing barriers to support. Taken together, these factors create a situation in which both teachers and parents can be confused about the value of diagnostic assessment for individuals with dyslexia. If support is provided according to need and a dyslexia diagnosis has no value in planning intervention, then why seek diagnosis? This is a question we will address by engaging with parents who have made the decision to pursue additional educational support for their dyslexic children.

## 2 Dyslexia and Associated Characteristics

There are systemic and geographic factors contributing to the risk of poor reading outcomes in addition to ‘within‐child’ factors. There is widespread evidence that poor educational outcomes are more likely in particular geographic areas (e.g., Johnson 2020). Quinn and Wagner (2013) found that rates of dyslexia identification were gendered, with 1:4 boys being identified with literacy difficulties but only 1:7 girls. Dyslexia is also less likely to be identified in some groups such as Afro‐Caribbean and Gypsy/Romany/Traveller heritage communities (Lindsay et al. 2006). In other words, cultural and contextual factors play an important role in how dyslexic students are identified and supported. Therefore, while much of the previous research on dyslexia has focused on the mechanics of learning to decode and spell, factors predicting the outcomes of individuals with dyslexia are much more complex, and it is crucial to consider the context in which a child is learning and developing.

Literacy difficulties are associated with wellbeing risks, such as increased anxiety, and some clinically recognised mental health difficulties (Carroll et al. 2005; Francis et al. 2019). The extent to which the association between dyslexia and mental health issues is explained by shared risk factors (such as genetics) or reciprocal causation, rather than directly as a consequence of dyslexia, remains unclear (Carroll et al. 2005). However, qualitative studies seem to suggest that mental health issues are caused by difficulties managing dyslexic needs in schools (Carroll et al. 2005; Dahle et al. 2011; Ingesson 2007; Kalka and Lockiewicz 2018; Leitão et al. 2017; Riddick 2010). Recently, Wilmot et al. (2023) identified the impact of fatigue and exhaustion at school on dyslexic pupils' mental health. Any failure or delay in recognising children with reading difficulties at school is likely to compound such risks, generating psychological stress.

Longer term, individuals with dyslexia are at increased risk of multiple negative social outcomes (Aro et al. 2019). Further work by Banks et al. (2023) has highlighted the cumulative social burden, increasing across the lifespan, that disability and inequality create. However, such research has had little impact on practice. The tacit assumption has been that effective skills‐based remediation of literacy difficulties will also address such secondary impacts, but we argue that this may not necessarily be the case.

When considering the impact of dyslexia in a classroom situation, it is crucial to consider whether the child with dyslexia experiences ‘inclusion’. Göransson and Nilholm (2014) have provided a useful four‐level descriptor of different levels of inclusion in a classroom/school, as follows:

- Inclusion as placement in mainstream classrooms
- Inclusion as meeting the social and academic needs of pupils with disabilities
- Inclusion as meeting the needs of all pupils
- Inclusion as the creation of communities.

The current study used this framework of different understandings of inclusion to frame the experiences of the pupils, as recounted by the parents.

## 3 Valuing Parental Accounts

Previous research that has addressed school experiences of dyslexic learners has tended to focus on personal experiences recalled by adults (cf. Deacon et al. 2020) or teacher reports (cf. Dymock and Nicholson 2023). Relatively few studies have examined parental narratives of children's progress over time. Parents and primary caregivers have unique perspectives on their children's experiences because they hold a longitudinal account of educational difficulties from preschool onwards. Moreover, they are consistent observers of the consequences of teaching, learning and the school experience on their child. They also bring other insights, providing an account of the lived experience of dyslexia beyond that observed in the classroom.

## 4 Rationale

The literature alludes to the realities for children with a profile of literacy‐based difficulties, and the impact of the contested definition of dyslexia (cf. Department for Education 2021; Elliott and Grigorenko 2014; Gibbs and Elliott 2020). However, there has been no work to date that has looked at the impact of how a focus away from dyslexia diagnosis and its core constructs (see Snowling et al. 2020) has impacted children's, parents' and teachers' experience of securing good outcomes and inclusion for children with written language difficulties. This study set out to understand the impact of securing or not securing a diagnosis of dyslexia on outcomes and inclusion for children and young people, using parental and teacher interviews as data sources. Parents were asked to share their stories of securing educational support for their children's literacy difficulties. Teachers were asked to talk about how they approached supporting children with dyslexia or other literacy difficulties in their classrooms. By interrogating these accounts in parallel, we were able to explore the role of dyslexia diagnosis in current educational practice, and how variations in that practice impacted children's outcomes.

## 2 Methodology

The focus of this study is on children in mainstream provision in English schools. In order to interrogate different perspectives in the same timeframe, parental accounts were analysed alongside accounts from class teachers and teaching assistants. This captured differing perspectives that could be used to support or challenge the other group's data and give a robust account. Data were collected from five Local Authority areas, and participants were recruited through snowball sampling via initial contact with knowledgeable others; this approach enabled the sampling of diverse socioeconomic/environmental characteristics, structural/policy frameworks, and activism levels. Participants were recruited in two phases: Phase One (2013/2014) and Phase Two (2017/2018).

An initial participant group of five parents was recruited through personal contacts; their children started in mainstream primary and all had Statements of Special Educational Need (later converted to Education Health and Care Plans) with literacy difficulties as a key feature. At transfer to secondary school, these parents went to tribunal to secure enhanced provision for secondary education including additional teaching and therapies. Intermittent contact was maintained with this group, and the pupils who remained as state sector pupils were followed up in Year 11 as part of Phase Two. In Phase Two, the teachers and remaining parents were purposefully recruited via snowball sampling in order to bridge gaps in the sample's diversity. Teachers from schools representing diverse socioeconomic profiles were recruited through key contacts to provide a contemporaneous account of mainstream classroom practices and understanding of literacy difficulties. With the exception of the rural primary school, these teachers did not teach the specific children belonging to the parent participants.

Most previous research on dyslexia has focused on one perspective from one group of respondents. In contrast, Critical Realism (Fletcher 2016) is a philosophical stance that allows multiple respondents' viewpoints to be synthesised. This is crucial to fully understand how dyslexia is understood in English schools; including the core issues of why outcomes for these students have been characterised at the end of education by persistent underperformance (Alexander‐Passe 2015; All‐Party Parliamentary Group For Dyslexia and other SpLDs 2019; see also Elliot Major and Parsons 2022). Fletcher (2016) converts the philosophical stance of Critical Realism into a research strategy. The two key methodological tasks are to elicit the observable/partially observable realities (the ‘actual’ level) and to identify the unobservable realities (the ‘real’ level). In order to do this, participant accounts (the ‘empirical’ level) are interrogated by the researcher and placed within the context of the other accounts to identify key evidence and drivers. The real level is achieved via a process of abduction (theoretical redescription) and retroduction (focusing on inferred causal mechanisms). Retroduction relies upon taking a critical stance and considering competing explanations (Yin 2018). For example, the impact of a school's acknowledgement of a dyslexia diagnosis, and their policies around assessment are the ‘actual’ level implied by the empirical level data of parent and teacher accounts. Analysis further allows for identification of ‘real’ levels themes such as ‘being able to see dyslexia’.

Our analytic process drew on reflexive thematic analysis (Braun and Clarke 2006). All the way through the analysis, consideration was given to the way that people told their own stories. At times, the words alone did not convey the emotion behind the story, and thus it was important to consider the narrative retelling in addition to the objective events. Annotations were used to capture the first author's responses to content using the facilities within NVivo and to avoid the risk of post hoc fallacy as well as check for rigour. In this respect, the approach is consistent with a reflexive stance as described by Braun and Clarke (2023). Another part of the quality process was a panel of four colleagues, each drawing from different methodological traditions and focuses, who acted as critical advisors during the analysis. These panel meetings were recorded and then used for further reflexive work.

### 2.1 Positionality

We positioned our work and analysis within a developmental and critical realism paradigm because the manifestation of dyslexia and subsequent life outcomes were seen as the interaction between core features within the individual and wider systems and environments. The mediating factor was the form and nature of agency in how individuals sought to manage or overcome structural features. This applied to children, parents and teaching staff. Critical realism does not take a position with respect to attributing causality to either biological or social–structural mechanisms, but rather takes the view that all forms of structure are contributory, and agency impacts upon their manifestation in the observable world.

Key to this at all levels was both the cognitive and social structure and what agency was exerted to manage structural impact. An additional dimension of positionality was that the principal impact of dyslexia was in the disturbance of inclusion and agency rather than just a focus on reading and writing. The first researcher's positionality was also informed by her own experiences of dyslexia and academic enquiry, as well as her experience working in the field of mental health and rehabilitation. This was salient when, as it transpired, she was dealing with parents who demonstrated evidence of trauma.

### 2.2 Interviews

The development of the interview protocols was based upon the work of Qu and Dumay (2011) for issues of balance and power, and Alvesson (2003) who developed a continuum of interview orientation and positioning from objective reality (positivist) to experiential (romanticist) with a mid‐point of ‘localism’ framing of both factual and experiential interviews. These were important, as the nature of the interview orientation would frame the quality of responses, with some strategies more likely to elicit structural features and others more agency aspects.

Two different semi‐structured interview protocols were developed. The interviews contained a mixture of semi‐structured and open‐ended questions, with content developed as part of a themed framework. Parents' interviews were framed around ‘tell me what happened in your experiences of getting support for your child’. For teachers, the theme was ‘what do you do to support individuals with dyslexia or literacy difficulties and why?’. In addition, all participants were asked ‘how do you understand inclusion and dyslexia?’

### 2.3 Participants

#### 2.3.1 Parents

There were 19 parent participants in the study (see Table 1). Parents had at least one child with dyslexia, with the ages of the children ranging from Year 2 (age 6–7) to Year 11 (age 15–16), and a cluster of 10 children at Year 7 and Year 8 (ages 11 to 13). Pseudonyms are used throughout.

**TABLE 1:** Parent participant details.

Name of parent in study	Name of their child(ren) in study	Level of education at time of interview	Interview format
Ann	Andrew	Secondary	Individual
Beth	Bob	Secondary	Individual
Cathy	Colin and Clare	Secondary and primary	Individual
Diane	Dave and Debbie	Secondary and primary	Individual
Elliot and Elizabeth	Ethan and Emma	Secondary and secondary	Joint
Gemma	George	Primary	Small group with teachers
Karen	Kevin	Secondary and primary	Individual
Lucy	Larry	Secondary	Individual
Nora	Nathen	Secondary	Individual
Oliver	Owen and Oscar	Secondary and secondary	Small group
Penny	Peter	Primary	Small group
Rachel	Robert	Primary	Small group
Susan	Sarah	Secondary	Small group
Tracy	Thomas	Secondary	Small group and individual
Vera	Vince and Violet	Primary	Small group
Wendy	Wayne	Primary	Small group
Xavier	Xara, plus two brothers	Primary	Individual
Yvette	Yves and Yasmin	Secondary and primary	Individual

#### 2.3.2 Teachers

Anonymised details of the teachers who were interviewed are detailed in Table 2, alongside the nature of the school they were working in at the time of their interview. Interviews with this group were undertaken either individually or as part of a small group. Schools ranged from urban schools with relatively high socioeconomic challenges, through to more suburban and rural schools.

**TABLE 2:** School staff demographics.

Name	Level of work experience	Type of locality of school	Types of school	Interview format
Frank	Senior experienced	Urban challenged SES	Large primary	Individual
Fraser	Experienced	Urban challenged SES	Large primary	Individual
Fay	Early career	Urban challenged SES	Large primary	Individual
Hannah	SENCO (Special educational needs coordinator)	Suburban	Mid‐size secondary	Individual
Hara	Experienced	Suburban	Mid‐size secondary	Individual
Holly	Experienced	Suburban	Mid‐size secondary	Individual
Jane, Jill, Josie, Jackie, Joan, Jennifer, Julia, Jasmin, Joy, Jenna	Experienced teaching assistants	Suburban	Mid‐size secondary	Small group
Gwen	SENCO	Rural	Smaller primary	Small group
Gabby	Experienced	Rural	Smaller primary	Small group
Gracie	Early career	Rural	Smaller primary	Small group
Imogen	SENCO	Urban: Challenged SES	Mid‐size primary	Individual

### 2.4 Data Collection and Analysis

Ethical permission was sought and granted from Coventry University Ethics approval system. Participants were recruited through word of mouth, via specialist teachers, and through community organisations. Interviews were undertaken by the lead author and were recorded, transcribed, corrected (as part of the familiarisation process), anonymised and then uploaded to NVivo, which was used to build analysis from the ground up. The value of NVivo for such a long project was that its tools allowed contemporaneous notes that were easily accessible and could be built upon in further analysis when returning to the data at a later point. In this respect, the data analysis was iterative rather than linear.

## 3 Findings and Discussion

**FIGURE 1:**
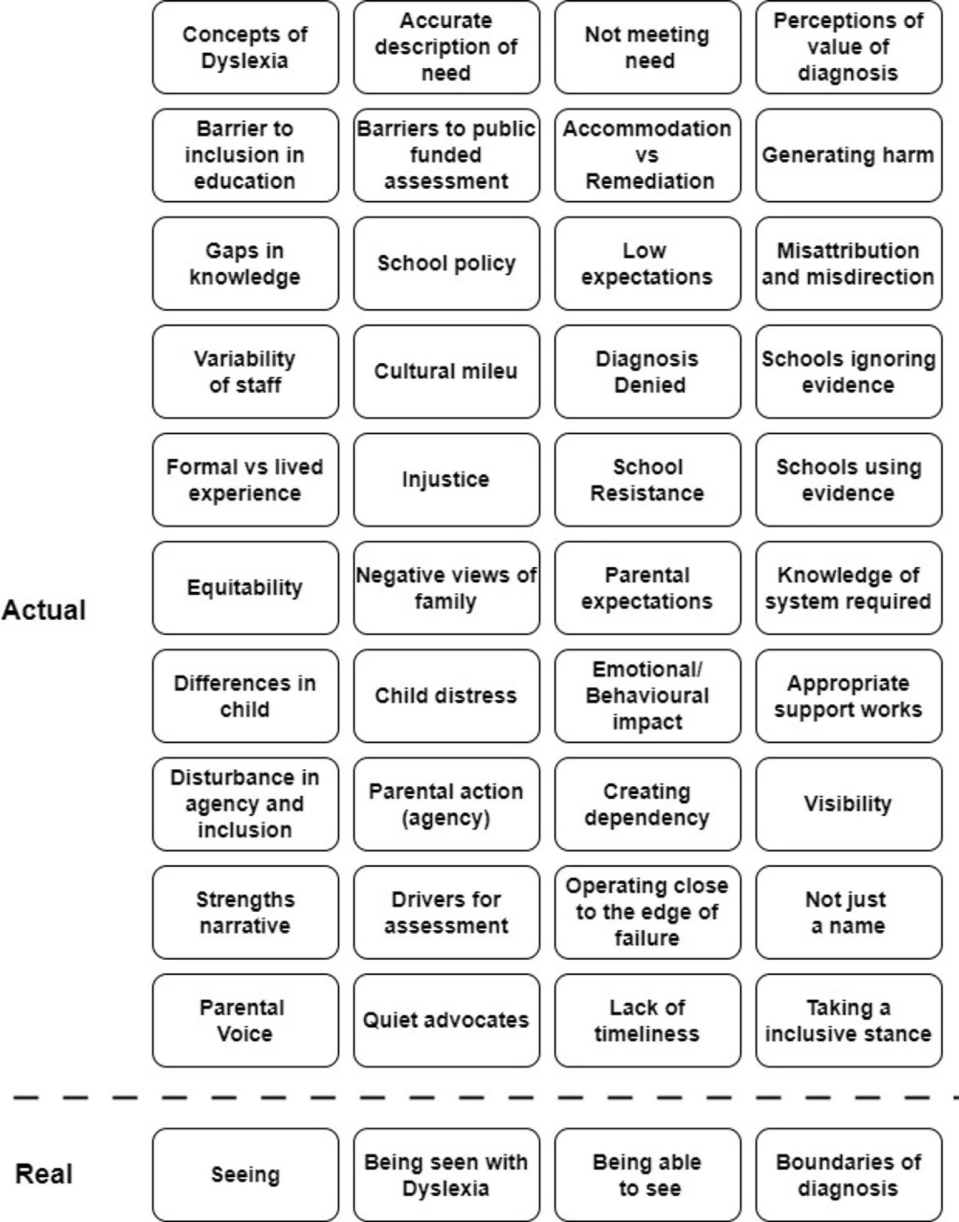
Simplified representation of subthemes identified at the actual level from data and the real level themes.

Our analysis enabled 40 subthemes at the actual level to be identified, each interacting with at least one other subtheme and most with multiple interactions. These are shown in Figure 1. Some connections facilitated visibility of the child and their needs across parents and school staff. They created positive ecosystems supporting, and thereby promoting, different levels of inclusion of meeting needs, a sense of community (Göransson and Nilholm 2014), and the child's access to education. Other combinations generated a negative ecosystem constraining or locking out a child's access to education and, in some cases, even dissolving basic level inclusion of having a presence in the classroom (or school) (Göransson and Nilholm 2014). There were also subthemes that were in conflict with each other. Many of the subthemes came directly from the voice of participants. Others were abstracted from patterns across the data.

Four ‘real level’ themes were extracted from the actual and empirical level data reported by parents and teachers. These were labelled ‘Seeing Dyslexia’, ‘Being Seen with Dyslexia’, ‘Being able to See Dyslexia’ and ‘Boundaries of Diagnosis’. In presenting the findings the parental voice has been foregrounded. This is consistent with the project's ethical stance.

### 3.1 Differences and Similarities Between Parent and Teacher Accounts

Some themes were much more prominent in parents' accounts and others more prominent in teacher accounts. Taken as a whole, parents and teachers agreed that some children require additional literacy support to succeed in school, but they differed in terms of how they felt it should be implemented. Narratives that centred on successful outcomes for the families tended to highlight synergies between parent and teacher perspectives, while less successful narratives often showed a disconnect between parent and teacher perspectives on the same issue. For example, teacher accounts tended to have a focus on short term concerns and the implementation of local policies, whereas parental accounts were more sensitive to the implications of their child's challenges over a longer period of time extending beyond a single academic year. Furthermore, parents were predominantly focused upon the socio‐emotional wellbeing of their child.

Broadly, while both parent and educator accounts agreed that problems in acquiring literacy gave rise to difficulties, the perspectives of the difficulties, the relative weighting of themes and the impact on the participants were different. Parents were predominantly focused on the impact on social‐, emotional‐ and confidence‐related outcomes for their child; on the distress that the educational experience and lack of peer‐equivalent progress gave rise to, as observed out of school and from the child's accounts of school. Parents also contextualised their account by considering the implications across the span of education and also post‐education. The use of longitudinal data was particularly helpful in this regard. It also enabled further identification of relevant data from the cross‐sectional interviews.

The lack of literacy progress impacted educational progress and was linked in many, but not all cases, to a lack of social confidence and for some to marked denigration or bullying. However, there were occasions where peer social connectedness and learning accommodations appeared to provide a protective mechanism. Under those circumstances, parents scaled back their efforts at securing literacy support, and the implication was that they did not see dyslexia as so problematic at that point. Importantly, social connectedness and accommodations did not address the fundamental problems of literacy difficulties. This did change when children were in secondary school, and the impact of unaddressed literacy difficulties became apparent to parents.

Teachers were principally concerned about progress at a group level, within the class year and the organisational impact of trying to secure that progress at the subgroup or group level, in the context of limited resources. This led to them using strategies such as putting strong students next to weaker ones in planning classroom layout or using combined class groupings at primary level to target learning. They were all aware of the need for children to make sufficient progress in their class. But there was no plan if the child failed to meet the target threshold. The knowledge base on dyslexia and effective management for educators was limited unless they had engaged in additional training. For some teachers, local education policy on the refusal to recognise the term or construct of dyslexia was an additional barrier to navigating effective management and access to specialist training.

These different perspectives are important for understanding how dyslexia can be rendered visible or invisible through the actions of teachers, schools and parents in the name of inclusion. They also illustrate both the shorter‐ and longer‐term implications of invisibility and visibility for children's academic and socio‐emotional outcomes.

### 3.2 Seeing Dyslexia: How Do Parents and Their Children View Their Lived Experience of Dyslexia?

Our analysis indicated that children with dyslexia and their parents did not see, or experience dyslexia as confined to a contextualised reading and writing deficit, as Elliott (2020) might suggest. Rather, they saw it as a source of disturbance in developing agency and inclusion across a range of settings, giving rise to distress. Their accounts often drew upon strengths‐based narratives. For example, as Oliver reported ‘somebody said to me [dyslexia's] a learning difference, it's a social difference, we're all different for many ways’. Similarly, Wendy's construction of dyslexia emphasised positivity: ‘I always big up dyslexia and he's very creative so I think he thinks it's all part of being creative’. Such understandings of dyslexia indicate a difference in the perspectives between traditional, formal definitions of dyslexia and the lived experience of it.

However, parents also saw dyslexia as a barrier to inclusion in mainstream education. Using the Göransson and Nilholm (2014) hierarchical model, their children were positioned at the lowest rung of being placed in the classroom but without needs being met. In the following exchange, Penny, Susan and Oliver, who had children in primary and secondary school, capture the themes that occurred across the data of how dyslexia impacted their children's inclusion in the learning environment, their capacity to express agency and the lack of awareness in teaching staff regarding learning needs. In this exchange Susan's demeanour and tone conveyed her anger at what she perceived to be a second‐class education her child received:

PENNY: Inclusion to me and the way it is with Peter is that they have, the educational system have set down, ‘and this is what they've got to be taught’, ‘this is the way they've got to be taught and at the age of this they've got to be able to do this, this, this and this’. Well, all children are different, and they don't all learn at the same rates. So, inclusion for me means that it's at the expense of his dyslexia, he isn't getting the teaching he needs and taught the way he needs to be taught in order to reach the same standard as them.

I: So actually, this idea of inclusion, everybody having the same actually acts as exclusion for your son?

PENNY: Yes.

PENNY: He's not being included because they have to be taught that way.

SUSAN: They're always given a classroom assistant.

OLIVER: Can I add to that, my wife has to play dumb me down because I've got very animated about it, very frustrated in that if my child had two legs missing and the PE teacher told him to get out and run the 100 metres, we'd be taking them off for discrimination. Yet Oscar and Owen, I hear time and time again, it's always from the sodding English department, ‘you're not trying hard enough’, ‘you can't read’, ‘you can't this’ and you think well yeah, he's got dyslexia.

The exchange above is illustrative of a how a limitation of visibility for a child's profile, including needs and strengths, generates environments where inclusion does not extend beyond the base level of presence in a class as described by Göransson and Nilholm (2014). It captures the degree of frustration and distress for the parents, linked to the lack of visibility of their child. The three parents explain that their children have been offered a lesser education and experienced exclusion or a lack of inclusion in different ways by non‐adapted curriculum delivery, being allocated to support staff rather than qualified teachers, and unsympathetic expectations and child blaming. It highlighted how the lack of skills meant a child's personal agency was compromised. In the case of Oliver's children, poor outcomes were attributed to a lack of effort on the children's part. For Penny and Susan, the isolation occurred because the child was either limited or unable to access education and express their agency as it was delivered to the majority of the class. This illustrates the complexity of sources of progressive failure in educational access with wide ramifications not only to inclusion in the here and now but the longer term too.

What parents frequently (but not always) saw was a mixed picture of uneven access to education across the years, poorly informed staff, combined with a policy that lacked sensitivity and responsiveness. Our data showed that this was apparent from the start of education:

TRACY: there appears to be no money, no specialist knowledge, not even a basic knowledge, I've had people …I had one teacher say I've been teaching for 15 years and I've never come across a dyslexic…

WENDY: the only frustration is recently in the last six months, with his weekly spelling test, which he fails every week because he can't possibly ever pass.

The extracts above capture how lack of knowledge and inflexibility generate progressive harm. The school in Wendy's case spotted there was a problem in Years 1 and 2 but then said they had to wait until he was 2 years behind before they could assess and meanwhile carried on without adaptions and were still doing so at the time of interview in Year 4. This is inconsistent with the SEND Code of Practice of Assess, Plan, Do, Review (Department of Education and Department of Health 2015) and is discredited practice, but also reflects a lack of knowledge and sensitivity.

Some years, or subject teachers, were better than others. There were examples of good practice and well‐informed staff in the data, with both staff and parents recognising this. In those accounts, there were examples of not only a child's needs being met but also a sense of community being engendered, thus accelerating the child up the inclusion hierarchy as described by Göransson and Nilholm (2014). One example was a rural primary school where the staff had received Local Authority training on dyslexia, and each of the three staff in the group interview had significant postgraduate training and qualifications. Their accounts demonstrated close working with parents and a critical stance on evidence‐based practice, policy and delivery of education:

GEMMA: He is eight [talking of George]. When he started school, I gave the teachers then the heads‐up. Then he went to the next teacher, and it's followed‐on from each teacher!….’ I got more information from the school this morning than I got last night, even then I didn't… he doesn't give that out freely

GWEN: we've made school‐based decisions which aren't always easily or happily…done, because training's taught people who teach children phonics and they learn by phonics, and they just keep plugging away at it until eventually one day they get it. Well, that is not the case, so we decided that Year 3, no, because that's just making them feel worse…. you've got to try and just prove it and doing the precision teaching.

GWEN: Yeah, we've got family learning, keeping up with children's English, that started on Friday.

GEMMA: Yes, so I came to that, so I could help George at school, so we're just learning verbs, nouns and things, that I'm meant to concentrate on.

In the above extract, the mother of George, Gemma, talks about how she liaised with the school on her son's needs, and finds out on a daily basis what he has been up to so she can engage her son at home and vice versa. There was evidence as illustrated above of reducing home school barriers and working in the child's interests. Gwen, who is the SENCO (Special Educational Needs Coordinator) discusses how her professional observations about the outcomes from a pure phonics program led her to investigate other teaching strategies that she identified by author, such as precision teaching and real book reading (Solity and Vousden 2009). This was supported by the other staff in the interview. Finally, Gwen and Gemma talk about a parental literacy engagement program. One observation from this interview was how staff drew upon their postgraduate training to articulate their positions, and this was unusual within the study.

In another interview Fraser, a teacher in a large urban school, also illustrated the value of diagnosis in facilitating the recognition of other pupils with written language difficulties as dyslexic ‘After teaching previous children who were diagnosed with dyslexia, I see similar traits with… I've got two girls in my class that show similar traits’. This led him to reach out to the family who it turned out had a history of dyslexia. This neatly illustrates how an individual child's visibility has wider ramifications for communities of learners which also include teachers and families elaborating the community aspect of the hierarchy (Göransson and Nilholm 2014). However, these types of informed practitioners formed a minority in the accounts.

### 3.3 Being Seen With Dyslexia: Barriers to Getting an Accurate Description of Need

Epistemic injustice (Byskov 2020) describes the forms of injustice that arise from those with relevant knowledge and information not being acknowledged or listened to. One of the challenges has been the positioning of parents as not holding relevant knowledge:

TRACY: Foundation, I was already talking to teachers going there's something wrong, why isn't learning at the same rate as everybody else?

I: And what were they saying back?

TRACY: He's a boy, just forget about it.

YVETTE: I went back to that teacher and said this is what I think [he had dyslexia], and she didn't really want to know, and whether that's a budgetary thing, they didn't want to go down the formal diagnostic route and that sort of thing, so we just left it really, and just tried to support him as much as we could.

XAVIER: I think for our little boy, we were not taken seriously. I think there was an undermining of the need.

The lack of accessible public funded diagnostic routes articulated in the data is also reflected in the literature (Knight and Crick 2021) and the reluctance of schools to commission assessments or have effective screening, placed very significant barriers for parents seeking support. In the extracts above parents were perceiving risk to wider inclusion due to disruption in learning progress, but this was not acknowledged. However, from a different perspective Imogen, SENCO in an area of deprivation, noted that for her area there were policy drivers and parental engagement factors around non‐acknowledgment: ‘because it's not diagnosed locally, parents have to pay for it privately if they want a diagnosis, [..] but parents just can't afford it. […] Yeah, I can't say it [assessment] wouldn't be viable for everybody but on the whole, it wouldn't be a priority as such’. One reading of Imogen's account is local cultural milieu and the localised narrative on the value of education may also act as a barrier for individual parents who want to support their child. Parents in the study sought diagnostic assessment because school provision is perceived to be failing their child, and they are seeking to increase the visibility and authority of their interactions as they try to secure support. In these respects, the parents value education, but what was also pervasive was they are also driven by their child's distress. For parents where there were difficulties in literacy and learning but the child was happy in school, they were not engaged in the same way. Beth talking about her son Bob in Year 3 was an example ‘that I maybe sat back on my laurels a bit because he was happy, he liked the teacher and he was making some progress, not vast amounts of progress…’. However, when Bob's distress and failure to make progress returned in Year 4 she took very direct action in securing support. Beth's account demonstrated the fluidity of inclusion levels (Göransson and Nilholm 2014) across years for this group of pupils, and the variable educational access. From this we see that because of the way inclusion is situated around a specific school year or classroom experience, discontinuities emerge as children transition from one class, teacher or year group to another. In this respect the accounts illustrate a limitation in focus of the Göransson and Nilholm (2014) account as it does not address the disruptive transitions between years and settings.

In many cases, it was child distress, rooted in lack of inclusion and loss of parity in agency, that led to parental action, often in the form of seeking formal diagnosis through private providers to make visible the child's needs and strengths. Tracy went on to describe what happened next ‘By the time he was seven, I'd borrowed some money off my dad to get him diagnosed as dyslexic which we did, that was Year 3, and they were still not interested, the teachers didn't seem to have any knowledge about dyslexia whatsoever’. Diverging from the narrative in Knight and Crick's (2021) account of the financially comfortable middle‐class securing diagnosis, the parents in this study came from a distribution of economic backgrounds. As Tracy alludes to, to secure the diagnosis she had to borrow money from family, others used credit cards or took out loans, and only some were able to afford it outright. But as Lucy noted the impact on non‐receptive schools was blunted ‘Oh yeah, he wrote a massive report but, because I'd taken it private, at that point in time the school wouldn't accept it because they hadn't done it’. Karen understood this rejection and persisted to get the school to pay, ‘I just felt, and I don't know if this is true or not, but I really felt I wanted it to come from the school. Because I felt I would be classed as some middle‐class mum whose child was underachieving and I just felt strongly that it would hold more sway if it came from the school’. One interpretation is that schools were resistant to making the child's needs explicitly visible at the individual level, as that was perceived to generate obligations.

A related issue regarding visibility and being seen, is that parental accounts of their children's experiences of dyslexia were minimised or denied. Yvette was unclear on why they would not investigate her son's needs, and this form of epistemic injustice was painful, given the obvious nature of his difficulty. It did not occur to her, or indeed the other parents in the study, that the school may have taken a hostile view towards the diagnosis of dyslexia. For them it was incomprehensible why action was not taken, and they assumed that the diagnosis was perceived as valid by the school. Frequently the parents sought explanations that lay outside the school, delay was attributed to lack of money, as captured by Lucy ‘I genuinely think it was all to do with money. They've got too many kids that year that have been diagnosed with dyslexia’ or Yvette ‘I just get the sense that primary schools are so budget constrained and don't have the knowledge almost that … I don't know what to expect them to do really’. It was only rarely that parents challenged this unaffordable account and Oliver was a case in point:

OLIVER: We pushed very hard with the school. ‘Too expensive, too expensive’, ‘right where is it, we'll pay for it’, ‘no you don't have to’, ‘well then you do it’, ‘oh well maybe next year when we've got the budget’, ‘well you're either going to do it or not’. So it was meeting after meeting with the head teacher being a case of well you say he can do better, you've got to get on his side and help.

Given Oliver's account there is a sense of schools not wanting the information, not that they could not necessarily afford the assessment in the context of overall budget. There was a clue later in Yvette's account when a supply teacher took the opportunity at a parents evening to tell her he thought her son had dyslexia ‘and the other thing that the supply teacher did was say don't just concentrate your efforts within school, reach out and see what else is available to you’. It can be inferred from the account overall that the supply teacher had taken the measure of the school and was advising accordingly. The hostile view was also reflected in Imogen's account of local policy, which has been promoted in some academic and education settings (cf. Gibbs and Elliott 2020), but for parents who observe the difficulties, the distress, the loss of inclusion and agency for their child, and the failure of the system to deliver education, this ‘deny policy’ and academic position has little meaning or validity.

### 3.4 Being Able to See Dyslexia: Consequences of Not Getting Description of Need or Diagnosis

It has been argued that dyslexia forms part of a natural continuum of difference, and all that is required is the application of evidence‐based interventions to resolve the issues (e.g., Protopapas 2019; Snowling et al. 2020). The proposition relies on individual teachers and schools being able to accurately recognise the dyslexic profile of needs and meet those needs. As can be seen from the above section, this is an important gap in the strategy. In the following extracts, different components of this approach are considered. In the first case, Tracy, who had paid for private lessons out of school and had secured a private assessment, found that both the primary and secondary schools refused to acknowledge or respond to the relevant information. In the extract, she described the current situation of her funding out of school lessons for Thomas, who was in Year 7, and the demands of schoolwork:

TRACY: I don't know because it's just a gut feeling, he's doing letters and sounds through the ‘Beat Dyslexia’ but he's been asked to critically analyse, he's doing ‘The Boy in the Striped Pyjamas’ and it's so much more advanced than just reading, you just think the gap is just getting bigger and bigger [..]

I: Can he actually read?

TRACY: He can read but he can read at the age of about a 6‐year‐old. [..] he certainly can't critically analyse it, he can't do anything to do with things like images and things like that it's way beyond him, he can't do it.

In this respect the diagnosis had no impact on successful inclusion for Thomas, but only because the school rejected it. In rejecting the information in the report, they also failed to address needs, or call in specialists to assist. It seems the school had low aspirations for Thomas, who was bullied, sometimes very badly, and despondent at school. He was isolated both socially and academically, despite his mother's best efforts.

The other aspects of Tracy's account that permeated the data were how parents saw dyslexia, and in particular the reading and writing aspects. They frequently commented on their child's difficulties with writing and spelling, which were self‐evident. However, reading difficulties, including age‐appropriateness, were less consistently identified by parents. This gap in understanding reading deficits was apparent across the data. Susan, who did persuade her primary school to get her daughter Sarah assessed when she was 10, recounted ‘We kept asking the school about it and they would just say oh she's just a bit behind. I kept saying to the school that I thought she was 12 months' behind where she should be’ but this assessment was only after an external tutor advised her to get a formal assessment, and she had additional supporting evidence:

SUSAN: all along I said I thought she was a year behind where she was, but she wasn't, she was two years behind and they said they'd never come across a child with such low self‐esteem. Normally if you read a report, it sounds better and you can pick more things out, more positives but the more ‘I read it, the worse it sounded’. [..] It did take quite a while then, it's not until she's now at secondary school (age 12 Year 8) that they're doing something about it.

The school failed to acknowledge Sarah's difficulties, despite Susan's prompting from the beginning, and had minimised rather than investigated. By the time of the assessment the damage was significant, and when information was provided towards the end of primary education, the school did not action the information in a timely and relevant way, and it took time in secondary school for help to be provided. This represented lost years of at best partial educational access and contingent impact on mental health. It also further illustrated one of the limitations of the Göransson and Nilholm (2014) hierarchical model which was derived from systematic review of literature, in that it is essentially focused upon the classroom. It speaks less to the transitions and future needs across the span of education and post‐secondary education.

Karen was in the same position as Susan; she had secured an assessment just prior to school transfer. In her case staff had been explicit about dismissing the value of diagnosis, which they delayed for 18 months; ‘They said, he doesn't need to be assessed because we've put everything in place as if he was dyslexic’. However, the value of this analysis, the confidence in their ‘diagnosis of need’ and their provision was rapidly debunked when Kevin transferred to his next school:

KAREN: He couldn't copy off the board or if he did he couldn't read it back to himself. Or he couldn't finish off writing what was down from the board there. So, they got someone else to write it for him. [..] now, [age 13 Year 8] they have just done some reading and he is at a reading age of eight and he is thirteen. So, he is very far behind. And that really, actually, I wasn't even expecting that this year. I didn't realise he was as far behind as that. So, that shocked me this year.

It was not clear that the secondary school had accessed or understood the diagnostic assessment, the key bridge to visibility which Karen had pushed for, or that the staff understood the implications and advice. The experience was demoralising and distressing for Kevin, and also demonstrates how failure to properly identify and address need becomes disabling. Such superficial approaches to intervention in the form of accommodation in situ had long‐term impact, and created dependency at points where expressing independence and agency was a key social task. From the accounts Kevin could be inferred to be a child with good cognitive potential, but Karen revealed he was on track for grade 3 in his GCSE's (GCSE's are qualifications taken at age 16 at the end of secondary education, a pass being grade 4).

It was unsurprising, given the lack of autonomy and acquisition of age‐appropriate skill, that when environmental adaptions and sensitive teaching were removed by a change of teacher there was a rapid deterioration in Kevin's behaviour. Karen had previously noted in science ‘He was getting incredibly good grades’ and wanted to be an architect. However, she went on ‘He had a new teacher, and I had a call in the first week. Kevin was mucking around and not settling down and I was really shocked because he likes science and he felt he was good at it’. This account was useful in highlighting how vulnerable to relatively small changes in environment children with the dyslexia and neurodivergent profile are, and the degree to which they are out of step with their peers' level of agency and skill and the difficulty it causes them.

The interface between the child with their profile of differences and history, the environmental demands, the context and their combined impact on agency (including public agency) was an important analytical construct. Within this, the role of diagnosis was salient, and the lack of or non‐recognition generated adverse outcomes. This was exemplified by an exchange between Wendy and Vera, parents of 8‐ and 9‐year‐old children:

WENDY: Some people have teased him. There was one issue that I was furious with the school about because they had to go up for school councillor and they had half an hour to write something and stand up and read it out.

I: They all had to do it?

WENDY: No, you could volunteer and I couldn't believe that Wayne volunteered for it. He wrote it out and stood up and couldn't read his own words and everyone teased him. [..] he came home and sobbed [..] that was a big one. I was really cross with the school.

I: Poor poppet. […]

VERA: Vince wanted to go up for school council but as soon as he realised he had to write a statement, he said no.

It was seen in the data that if schools and their staff do not recognise dyslexia and do not accept the value of diagnosis (which this school apparently did not), the policies of inclusion and education become meaningless for children with the lived experience of dyslexia. But they also become a source of difficulty and stress for the family. Susan talked about the strain on her marriage as a result of Sarah's distress expressed at home and Karen recounted ‘his behaviour at home was awful as in tantrums, throwing himself on the floor, storming off and I've had to really work at that and be patient. Me and my partner have had a few squabbles over how to deal with that’. Schools are at risk of generating disabling and distressing environments. Even young children aged 6 were reported to physically withdraw, disengage and protect themselves from social damage.

It was not just pupils who withdrew, but also parents who recognised how hard and expensive dealing with a school or Local Authority could be; as Imogen commented, local policy would add another layer of difficulty. It is not surprising that it was only the most resilient of parents who secured support; other parents just gave up. In the extract below, Yvette, who had very challenging times with the school for her son (for whom she had been able to afford a diagnostic report), revealed that although her daughter's presentation of dyslexia was worse than her son's, no diagnosis had been made. The school had offered an assessment that she had ‘dyslexic tendencies’ (this was a term reported by many parents), but parental circumstances had changed, and she was not in a position to pay for support or endure that form of interaction again:

YVETTE: I don't really know, I've almost written off her primary years because from speaking to other parents everybody just says you'll see a massive change when you go to secondary school which is what's happened with Yves, and everyone says you just do not get the support in primary school, you've just got to get through it and then hope that things change when you get to, it sounds really defeatist when you actually say it out loud but I'm not, I'm just not expecting a lot from them.

### 3.5 The Boundaries and Utility of Dyslexia Diagnosis

Our analysis supports the interpretation that a failure to diagnose leaves substantial ambiguity and risk of harm across a child's education, leaving open the possibility of misattribution and misdirection. It supports the case that the visibility of the child and their needs is critical to ensuring effective intervention. It further supports the claim that the absence of detailed assessment by knowledgeable professionals is a barrier to avoiding preventable disability. However, for effective inclusion beyond the base level as described in the Göransson and Nilholm (2014) hierarchy to occur, staff need to be able to make use of assessment report information and also recognise the profile of strengths as referenced by parents in their account of the profile. This is also implied by the top of the hierarchy as a form of community by Göransson and Nilholm (2014). Dyslexia severity from these data appears significantly impacted by what happens between school and family. However, a diagnosis on its own does not resolve the issues; it is a tool to facilitate the process and recognition by the child, family and school.

The value of dyslexia diagnosis lies in the acknowledgment of it by those who can act on the information. In the case of Larry, who threw a chair across a classroom in frustration 5 months into his new school, his mother had paid for an assessment and had a diagnosis. The primary school ignored it until there was a change of SENCO. When he transferred to secondary school, she gave the report to the new school and met with the SENCO to discuss the support Larry needed. When Lucy followed up on the incident, she checked on all the support she had thought was in place ‘So then I said to her ‘so do all of his teachers know that he's dyslexic?’ ‘He's not dyslexic’. ‘Okay’ [said with alarm]. If you typed his name in to his student profile it just came up with a little picture of him, it didn't have anything, nothing’. Despite the meeting with the SENCO, the school had ignored the report and practical suggestions.

Although being able to name the difficulty allows for easy visibility, the account above outlines how important knowledge of the system is to ensure that legally required provision of support is applied. What matters is the evidence in the report and the education/legal system being activated via the SEND Code of Practice (Department of Education and Department of Health 2015), not the name the profile is given. Yet many parents did not appreciate this, and schools and others with responsibility did not enlighten them. Oliver was a typical example:

OLIVER: So, we got that done and a statement done, went into secondary school.

I: So he actually got a statement.

OLIVER: Yeah you get a report back don't you, not a statement sorry, a report back with recommendations in there. He had, I can't think what you called it, the learning plan. Owen had that learning plan.

Oliver did not appreciate this had no legal weight and could not be enforced, and all that was offered was extra time in exams. As he recounted earlier, teachers still did not address observed need for his son and his emerging disability.

The data contained many accounts of avoidable disability with long‐term consequences. Some of this was very distressing to record, including details of an 8‐year‐old child attempting suicide, but fortunately not knowing how to do so effectively. In that case, the parent could not afford assessments, so need had not been properly described and defined, and in this ambiguity, it can be inferred that the school was taking a negative or even hostile view of the family. The impact on the child and parent was significant. The school refused to acknowledge parental concerns. There were observably marked literacy and learning difficulties and other co‐occurring difficulties. It was only later when the parent was able to access assistance to submit a request for a statutory needs assessment that the nature and extent of the child's difficulties were articulated. This led to a change of primary schools and improved provision. However, the proposed secondary school were resisting provision, so that the parent had to take the local authority to a tribunal, where they secured the help required. To do this, the parent had to pay for a full diagnostic assessment. With this latter assistance in place the young person did well enough to enter an apprenticeship program. The case highlights both the failures and damage that occurs when quality assessment is not undertaken and the positive impact on a young person's life trajectory when diagnosis and support are applied.

## 4 Concluding Thoughts

Looking at the themes and subthemes identified, it is unsurprising that the problems dyslexic children have are often unresolved through education. The work of Thompson (2021) laid out the complexity of the structural features and agency that shape the expression of dyslexia a child has. A critical part of that account was the level of visibility the children and parents had. In this paper, the issue of visibility has been considered in the context of a prevailing narrative of non‐recognition and non‐diagnosis. The wider subtext in the data is of how inclusion is perceived by educational systems and schools as reducing individual visibility and having a generic accommodation inclusive strategy. In particular, it has considered the harm inflicted on pupils and families with this approach. In the literature and in practice, it is argued that the first line of diagnosis is teacher assessment and intervention (Fuchs and Fuchs 2006; Lynn et al. 2015). This should be instigated at the first opportunity with quality interventions such as phonics (Department for Education 2021). A child's responsiveness to good quality intervention can provide important information as to the nature of their difficulties (Rose 2009). In some cases, appropriately intensive and well delivered early intervention can even avoid the need for full diagnostic assessment. However, even in an environment where high quality literacy tuition is the norm and intervention is provided at an early stage, there will remain some children with significant difficulties, and our investigation has indicated the significant negative consequences associated with minimising these difficulties. Further, one of the persistent findings across the data was the variability in teacher expertise to deliver on this strategy, leaving parents and children in a state of disarray. When parents did try to articulate their concerns, they often experienced dismissal, injustice and distress. Parental concerns have also been dismissed in the literature (Knight and Crick 2021; Lauchlan and Boyle 2020), which forms the academic narrative background of privileging teacher viewpoints, even with limited knowledge. The Göransson and Nilholm (2014) hierarchy does not speak to this narrow perspective. From the data, there was evidence where schools engaged with parents and the child's visibility was raised; the progress up the hierarchy of inclusion was more effective.

Parents sought diagnosis because their children were experiencing distress; literacy difficulties were an obvious focus and parents were looking for some form of explanatory framework which they could use to navigate the education system. Their expectations were that a diagnostic report would increase the visibility of their child's needs, and schools would then provide appropriate support for their child. They had poor appreciation of the support system and how to ensure the report translated into obligations of assistance. In that respect a diagnosis was a retort to the epistemic injustice they were experiencing. However, as noted previously and in Lucy's case, schools could simply ignore this. The analysis highlights that without good quality information typically found in diagnostic assessments and access to knowledgeable and responsive teachers, access to education was compromised. In school cultures that only consider surface needs and contextual adaptation without remediation, the lack of diagnostic information becomes a source of disability and disenfranchisement for the child with predictable potential negative lifelong consequences.

Within this context, the four real level themes derived from parents and teachers' accounts provide a framework for critical analysis of current education training, practices and policy, education delivery, impact and outcomes. Such visibility of the agency and structural features embedded and reflected in the real level themes and the actual level subthemes challenge the research and practice in the field as it currently is. This is with respect to both within and, importantly, post‐school life for a child, where reaching potential and inclusion is not classroom‐based but society‐based. In this respect, the work in the field of dyslexia has a wider impact for all children with Special Educational Needs. An important feature of this for children with a dyslexia‐type profile is early diagnosis, the value of diagnosis being recognised, and the mitigations at both individual and structural levels that can flow from this being applied.

It might seem from our data that there is an ‘unbridgeable gap’ between parent and teacher perspectives. However, we do not believe that this is the correct conclusion. Our argument is that the gap can be bridged, to a certain extent, by the effective and consistent use of diagnosis of dyslexia. In an imperfect system, a diagnosis acts as a bridge of common communication and understanding to make visible a child's profile of needs. This visibility is needed because of the multiple ways in which the dyslexic profile can impact upon life within and beyond school. We therefore argue there is an important role for diagnosis that goes beyond simply labelling a child.

## Ethics Statement

Coventry University Ethics approved this study (P16301, P32566, P32394, P61676).

## Conflicts of Interest

The authors declare no conflicts of interest.

## Data Availability

As per ethics approval, data are not available for confidentiality reasons. Data were only available to those approved by Coventry University Ethics Committee and included those directly processing the data, the supervisory group and the examiners of the original thesis as requested.
